# Analysis of genetic evolution and molecular transmission of hepatitis C virus in key areas of the southeast coast of China

**DOI:** 10.3389/fpubh.2026.1746631

**Published:** 2026-03-04

**Authors:** Zongqing Li, Shaobin Wu, Wei Liu, Yiqun Liu, Qiaoling Lian, Shouli Wu, Chunyang Zhang, Jianfeng Xie

**Affiliations:** 1 School of Public Health, Fujian Medical University, Fuzhou, China; 2 Fujian Provincial Center for Disease Control and Prevention, Fuzhou, China; 3 Fujian Provincial Key Laboratory of Zoonosis Research, Fuzhou, China

**Keywords:** characteristics of epidemics, genetic analysis, hepatitis C, molecular transmission network, phylogenetic tree

## Abstract

**Background:**

Hepatitis C virus (HCV) infection is prevalent worldwide. The genotype (GT) distribution characteristics and transmission patterns of HCV show regional differences. Currently, the research on HCV genotype characteristics, molecular transmission networks, and associated risk factors in key areas of the southeast coast of China remains limited. This district lacks accurate molecular epidemiological data on HCV, hindering the implementation of prevention and control efforts. This study aims to investigate the epidemiological characteristics and molecular network transmission features of hepatitis C genotypes in key areas of the southeastern coast of China, to provide a reference basis for prevention and control strategies.

**Methods:**

The HCV RNA-positive serum samples (88 cases) were collected from sentinel surveillance subjects with hepatitis C in Xiuyu District, Putian City, Fujian Province, China, from October 2022 to April 2023. The Core and NS5b fragments of the HCV genome were amplified by Reverse Transcription-Polymerase Chain Reaction (RT-PCR). The PCR products were sent to Sangon Biotech (Shanghai, China) Co., Ltd. for purification and Sanger sequencing. The sequences were assembled and aligned using biological software. The processed sequences were used to construct phylogenetic trees and molecular transmission networks. The Fisher–Freeman–Halton exact test was used to evaluate the significance of differences in demographic characteristics between HCV genotype groups.

**Results:**

A total of 59 sequence samples were obtained from October 2022 to April 2023 (53 in the Core fragment and 45 in the NS5B fragment). Phylogenetic tree analysis showed that the major HCV genotypes were 1b (36/59, 61.02%) and 2a (22/59, 37.29%). One sample showed inconsistent genotyping results between the two genomic fragments. Most HCV strains had similar genetic evolutionary relationships in the Xiuyu District. There was a statistically significant difference in the distribution of HCV genotypes across occupations (*p* < 0.05), whereas no other factors showed significant differences. At genetic distance thresholds of 0.018 and 0.043, Core and NS5B sequences formed the highest number of transmission clusters. A total of 40 sequences formed the molecular transmission network, with an overall clustering rate of 67.80%. The Core fragment contained 6 clusters, and the NS5b fragment contained 5 clusters. The connection between samples in the GT 1b transmission cluster was closer than in GT 2a. The network transmission figures reflected the potential connections between samples.

**Conclusion:**

In this study, the HCV genotypes in the key areas were predominantly 1b and 2a. Most hepatitis C strains shared a similar evolutionary relationship. It suggested that there might be cluster transmission in this area. However, we still need more detailed epidemiological case investigations and experimental data in the future to obtain an accurate estimate of hepatitis C prevalence. Providing targeted treatment for HCV-infected individuals and intensifying screening among farmers are crucial measures to prevent and control hepatitis C in Xiuyu District.

## 1 Introduction

Hepatitis C has become the second most common viral hepatitis in the world, and is an infectious disease caused by HCV infection (1). It is widespread and often leads to chronic liver disease, with severe cases progressing to cirrhosis or liver cancer. Without prompt treatment, only 15%–25% of acute HCV patients recover spontaneously; the remainder develop chronic infection. Studies indicated that around 20% of chronic cases advance to severe liver disease over 20 to 30 years, and these patients frequently experience extrahepatic complications (2, 3). According to the World Health Organization (WHO) Global Hepatitis Report 2024 (4), about 50 million people worldwide had chronic HCV infection. There are approximately one million new infection cases each year. Approximately 24,2000 people died due to hepatitis C in 2022, with the majority of these deaths due to HCV-related cirrhosis and Hepatocellular Carcinoma. In China, an estimated 7.6 million people were infected (5). WHO’s interim goal is to reduce viral hepatitis incidence to below 5 per 100,000 annually; however, China’s hepatitis C incidence stands at 14.38 per 100,000, which is well above this target (6). Unfortunately, there is now no effective vaccine for hepatitis C. Therefore, studying the molecular epidemiology and transmission patterns of HCV is important for improving prevention and control strategies.

HCV is transmitted primarily through bloodborne transmission, sexual contact, and mother-to-child transmission. Among these, bloodborne transmission is the most significant pathway, commonly occurring through the sharing of contaminated injection equipment (7). Before the advent of direct-acting antiviral agents (DAAs), the standard treatment for hepatitis C consisted of pegylated interferon (Peg-IFN) in combination with ribavirin (RBV). This regimen was associated with a relatively low rate of sustained virological response (SVR) and substantial adverse effects (8). DAAs regimens achieve SVR rates above 90% in patients with HCV infection (9). However, resistance-associated substitutions (RASs) can reduce antiviral effectiveness and even lead to treatment failure (10, 11). Resistance sites within the HCV genome are unevenly distributed across different nonstructural proteins. The Non-structural protein 5B (NS5B) fragment has more resistance sites than Non-structural proteins 3/4A (NS3/4A) and 5A (NS5A), making it more prone to resistance (12). Common resistance sites in NS5B included L159F, S282T, and C316N, among others (13, 14). Therefore, pan-genotypic DAA regimens are not all-genotypic regimens. For special populations of infected individuals, appropriate treatments still need to be determined through standardized clinical trials or genetic testing.

HCV is a single-stranded, positive-sense RNA virus of about 9,600 nucleotides. It belongs to the genus *Hepacivirus* within the family *Flaviviridae* (15). The HCV genome contains untranslated regions (UTRs) and an open reading frame (ORF) that encodes a polyprotein. This polyprotein yields three structural proteins and seven nonstructural proteins under the action of related enzymes (15–18). HCV genome sequences exhibit high genetic variability (19). To determine HCV genotypes or subtypes, researchers often use fragments of the NS5B, Capsid core protein (Core), and Envelope glycoprotein 1 (E1) genes (20). Phylogenetic tree analysis, a key bioinformatics method, assesses evolutionary relationships between species or molecules via sequence homology. Recently, researchers have also applied molecular transmission network methods to examine HCV transmission patterns and epidemiological history (21–23).

Currently, eight major HCV genotypes (GT1–GT8) and over 100 subtypes have been identified worldwide (24). Genotype distribution varies: GT1, GT2, and GT3 are global; GT4 is mostly in the Middle East and North Africa; GT5 is mainly in South Africa; GT6 is common in South Asia and Hong Kong, China; and GT7 is in Central Africa. GT8 was recently reported in India (25–27). In China, GT1b and GT2a are the most common genotypes, with GT1b predominating, accounting for approximately 56.8% of cases (9). Different genotypes affect pathogenicity and response to therapy (17, 28). Therefore, genotyping is essential for personalized patient treatment. In Guangdong Province, a study found subtypes 6a and 1b to be the most common among HIV/HCV coinfected patients. Transmission network analysis showed stronger links among unmarried people than among married or cohabiting people (29). In Huazhou City, a history of blood transfusion and residence in Shi Village were identified as high-risk factors in a transmission network analysis (23). Studying genotype distribution and molecular transmission networks in different regions reveals local epidemic trends. This approach helps identify high-risk populations and influencing factors, so targeted interventions can prevent further HCV spread.

Prior studies have mainly focused on the epidemiological characteristics of HCV in the whole country or a large region; meanwhile, there is limited relevant molecular epidemiological analysis for Xiuyu District, Putian City, Fujian Province. Based on our previous study (30), Putian City’s Xiuyu District, located on the southeastern coast of China, is identified as the most likely cluster area for Hepatitis C virus (HCV) in Fujian Province. Therefore, this study aims to further explore the genotype distribution, transmission patterns, and evolutionary characteristics of HCV among infected individuals in this district through phylogenetic analysis and transmission networks, and provide a reference for HCV prevention and control.

## 2 Methods

### 2.1 Study subjects

The serum samples were collected from sentinel surveillance subjects with hepatitis C in Xiuyu District, Putian City, Fujian Province, China, from October 2022 to April 2023 (a total of 1,300 cases). Sentinel surveillance antibody-positive subjects were tested for nucleic acid. The RNA-positive samples (88 cases) were transported to the Fujian Center for Disease Control and Prevention and stored at −80 °C. This study was approved by the Medical Ethics Committee of Fujian Provincial Center for Disease Control and Prevention. HCV RNA-positive samples that met the “Diagnostic Criteria for Hepatitis C (WS213-2018)” and had not received antiviral drug treatment were included in the study. Patients with recent anti-HCV treatment, RNA-negative, or those with combined other viral hepatitis were excluded.

### 2.2 Nucleic acid extraction, amplification, and sequencing

HCV RNA was extracted from 140 μL of serum samples according to the operation manual of QIAamp Viral RNA Mini Kit (QIAGEN GmbH, Cat. No. 52906, America). The two fragments of HCV Core and NS5B were amplified by RT-PCR using OneStep RT-PCR Kit (QIAGEN GmbH, Cat. No. 210212, Germany). Reaction conditions: 30 min at 50 °C, 15 min at 95 °C, then 35 cycles of 40 s at 94 °C, 40 s at 54 °C, and 40 s at 72 °C, followed by 10 min at 72 °C and holding at 4 °C. The primers are designed with reference to the literature (31). The primers were synthesized by TaKaRa Bio, Inc. (Beijing, China). Core fragment forward primers (5′-AGGCCTTGTGGTACTGCCTGATA-3) and reverse primers (5′-GATTGTACCCCATGAGGTCGGC-3′); NS5B fragment forward primers (5′-TGGGSTTYKCSTATGAYACYCGMTGYTTTGA-3′) and reverse primer (5′-ARTACCTRGTCATAGCCTCCGTGAA-3′). The 5 μL PCR product was subjected to 2% agarose gel electrophoresis to observe the target band. The amplification products were purified and sequenced by Sangon Biotech (Shanghai, China) Co., Ltd.

### 2.3 Phylogenetic tree analysis

The sample sequences were assembled, aligned, and corrected using SeqMan and EditSeq software to obtain the target gene fragments. The obtained sequences were aligned with GenBank sequences using the Basic Local Alignment Search Tool (BLAST) to determine the sample subtypes preliminarily. HCV international standard reference sequences were selected from GenBank (M62321.1, M67463.1, D90208.1, M58335.1, D14853.1, AY051292.1, KJ439768.1, D00944.1, AB047639.1, D10988.1, AB030907.1, D50409.1, JF735114.1). The sample sequences (the accession numbers: PX917207-PX917304) and the reference sequences were combined, aligned, and trimmed using MEGA 12.0 software. Phylogenetic trees were constructed using the Neighbor-Joining method with 1,000 bootstraps. The tree was then adjusted and visualized using the iTOL online platform.

### 2.4 Molecular transmission network analysis

The pairwise genetic distances between sequences were calculated using the Tamura-Nei 93 model in MEGA 12.0. The genetic distance threshold was defined as the value at which the maximum number of transmission clusters is observed within the molecular transmission network (32). Network files were generated using R 4.4.3 and then imported into Cytoscape 3.10.3 to construct the molecular transmission network.

### 2.5 Statistical analysis

The Fisher–Freeman–Halton exact test was used to evaluate the significance of differences in demographic characteristics between HCV genotype groups. Statistical analyses were performed using R 4.4.3 software, and *p* < 0.05 was considered statistically significant.

## 3 Results

### 3.1 Demographic characteristics of study subjects

A total of 88 RNA-positive samples were collected from October 2022 to April 2023. Ultimately, 59 valid sequences were obtained. Among these, 53 samples were sequenced for the Core fragment and 45 for the NS5B fragment. Most cases were female (64.41%), farmers (86.44%), and aged ≥60 years (84.75%). The geographic distribution of cases varied, with the majority concentrated in Pinghai Town (44.07%). The genetic subtypes of the samples were mainly 1b and 2a, accounting for 61.02% (36/59) and 37.29% (22/59), respectively. One sample showed inconsistent genotyping of its two fragments. The genotyping in the Core fragment was GT2a, and that in the NS5B fragment was GT1b. Statistically significant differences were observed only in occupational distribution by HCV subtypes (*p* < 0.05), while no significant differences were observed in other characteristics (Table 1).

**Table 1:** Demographic characteristics and genotype distribution of the study objects.

Parameter	Number (proportion)	Genotype [number (proportion)]			*p* value
		1b	2a	Others	
Age					
<60	9 (15.25)	6 (66.67)	3 (33.33)	0	1.000
≥60	50 (84.75)	30 (60.00)	19 (38.00)	1 (2.00)	
Gender					
Male	21 (35.59)	15 (71.43)	5 (23.81)	1 (4.76)	0.098
Female	38 (64.41)	21 (55.26)	17 (44.74)	0	
Occupation					
Farmer	51 (86.44)	28 (54.90)	22 (43.14)	1 (1.96)	0.029
Others	8 (13.56)	8(100.00)	0	0	
Place of residence					
DaiTou town	19 (32.20)	9 (47.37)	9 (47.37)	1 (5.26)	0.254
Nanri town	14 (23.73)	11 (78.57)	3 (21.43)	0	
Pinghai town	26 (44.07)	16 (61.54)	10 (38.46)	0	

### 3.2 Phylogenetic tree analysis

**Figure 1:**
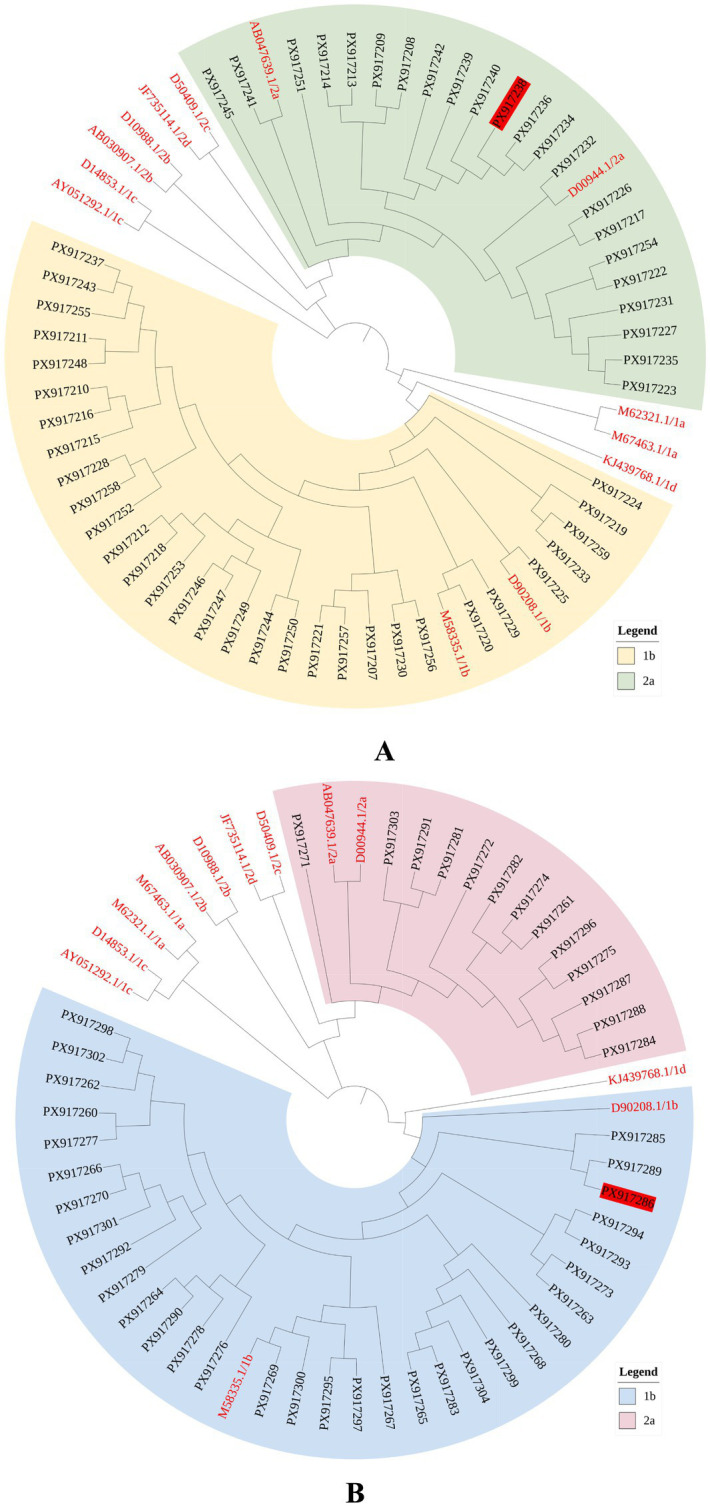
The phylogenetic tree based on the Core/NS5B fragment sequence. **(A)** Core; **(B)** NS5B. Sample accession numbers: PX917207-PX917304. Red font: reference sequence. The marked with a red background: sample with inconsistent typing in two fragments.

The phylogenetic tree showed that the HCV genotypes in Xiuyu District, Putian City, were 1b and 2a. In the Core fragment, 31 sample sequences clustered with the international standard reference 1b strain, while 22 sample sequences formed a cluster with the international standard reference 2a strain (Figure 1A). In the NS5B fragment, 32 samples clustered with the international standard reference 1b strain, and 13 samples clustered with the international standard reference 2a strain. Most sample strains were further subdivided into smaller branches and subsequently clustered together. The phylogenetic trees of the NS5B and Core fragments showed similar topologies (Figure 1B).

### 3.3 Molecular transmission network analysis

A total of 40 sample sequences were included in the molecular transmission network, with an overall clustering rate of 67.80% (40/59). The network clustering rates for subtypes 1b and 2a were 75.00% (27/36) and 54.55% (12/22), respectively. There were 13 samples with more than 5 edges in the molecular transmission cluster, of which 7 cases were 1b and 5 cases were 2a. Most of these highly connected samples were from individuals aged ≥60 years (10 cases).

**Figure 2:**
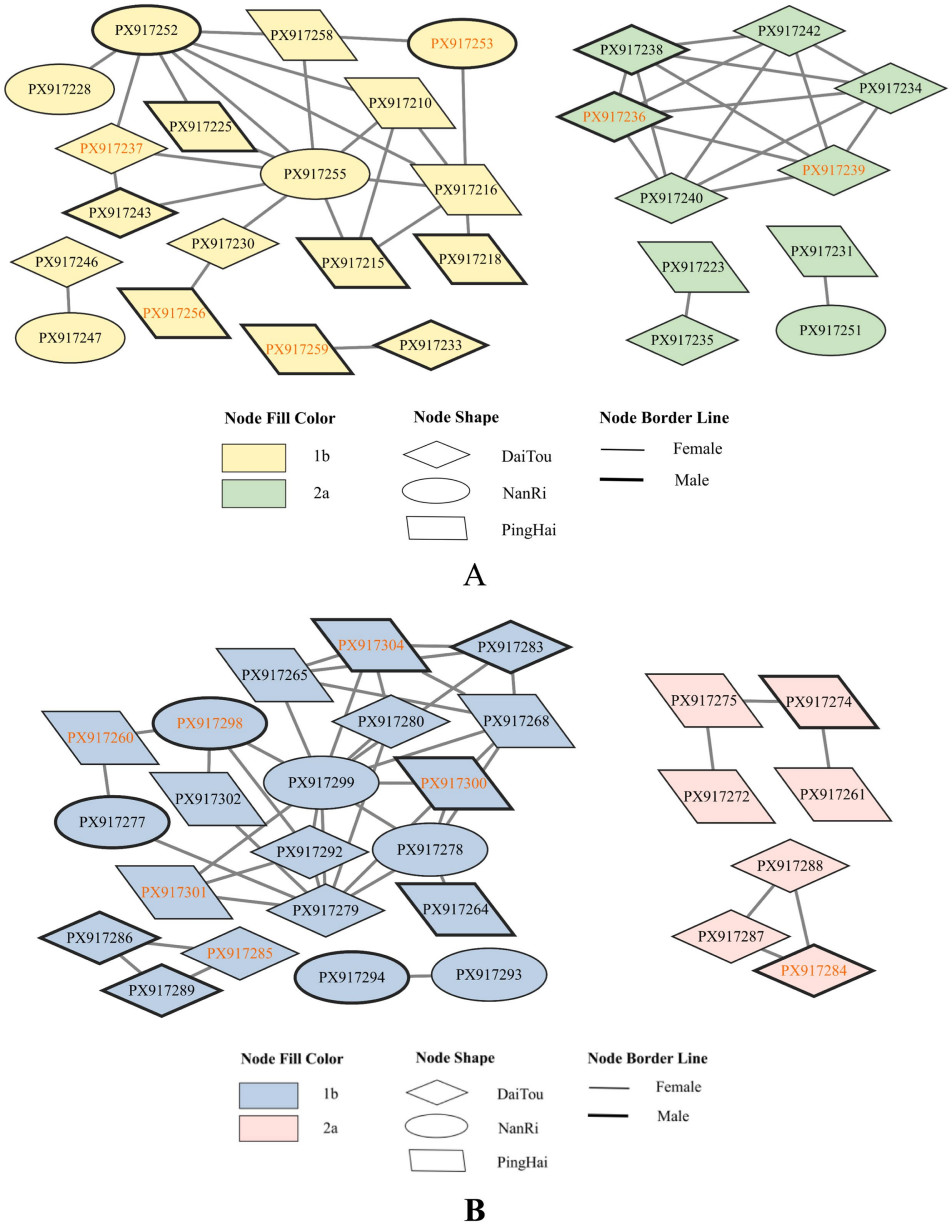
Identification of the HCV molecular transmission network based on the Core/NS5b fragment. **(A)** Core; **(B)** NS5B. Orange font: aged <60 years.

The genetic distance threshold was set at 0.018; the molecular transmission network in the Core fragment contained the highest number of transmission clusters. It showed 6 clusters, with a sample clustering rate of 52.83% (28/53). The largest molecular cluster consisted of 14 subtype 1b sample sequences. Within this cluster, the majority of cases (50.00%) from Pinghai Town and individuals aged ≥60 years (78.57%) were included in the network. The subtype 2a transmission cluster included 10 sample sequences. Approximately 90.00% of the patients in this cluster were from Daitou Town and Pinghai Town (Figure 2A).

The genetic distance threshold was set at 0.043; the molecular transmission network in the Core fragment contained the highest number of transmission clusters. It showed 5 clusters, with sequence counts ranging from 2 to 16. The clustering rate of the sample was 62.22% (28/45). 75.00% (12/16) of the infected individuals were from Pinghai Town and Daitou Town, and 68.75% (11/16) were aged ≥60 years in the largest cluster. There were 2 clusters of HCV subtype 2a. The infected individuals in Pinghai Town and Daitou Town formed separate clusters, with 85.71% (6/7) of them aged 60 or older (Figure 2B).

## 4 Discussion

The distribution of HCV genotypes shows certain geographical and population differences. Genotyping is valuable for analyzing HCV transmission routes and guiding genotype-specific treatment strategies (33). Xiuyu District in Putian City was identified as the area with the highest HCV infection rate and the most concentrated epidemic in Fujian Province (30). However, the understanding of the HCV genotypes and transmission patterns in this district remained insufficient. In this study, we conducted a molecular epidemiological analysis of HCV-infected samples. The results showed that subtypes 1b and 2a were the predominant genotypes. This finding was consistent with other studies on HCV genotype distribution (27, 34, 35), which reported that subtypes 1b and 2a were the predominant genotypes in Fujian Province.

Phylogenetic trees provide information about viral hierarchical relationships and genetic evolution (36). We performed phylogenetic analysis of the sample sequences from the two fragments. The genotyping results were consistent with those obtained from BLAST. Phylogenetic analyses of both genomic fragments showed that GT 1b strains clustered on one branch, while GT 2a strains clustered together on another branch. Most sample strains coalesced into distinct, smaller branches, which ultimately merged into a single overarching cluster. These findings indicated that most HCV strains in this district may share a similar ancestral genetic lineage and that familial clustering or localized transmission may be occurring. Future studies should combine family history and genetic data, followed by pilot studies in high-risk households to confirm transmission for targeted prevention.

The findings of this study showed a statistically significant difference in the distribution of HCV subtypes among farmers. The reason for this phenomenon may be that farmers lack knowledge of HCV prevention and control (37). Additionally, farmers generally have low health-seeking awareness, and HCV infection shows no obvious symptoms in the early stages, often leading to delayed detection or intervention. As a result, this subtype continues to persist and accumulate within the farming population. The study indicates it’s necessary to enhance health education on HCV prevention and control for farmers while also increasing screening efforts in this population to facilitate early treatment and intervention.

Previous studies indicated that unsafe medical practices, blood transfusions, vertical transmission, and needle abuse were associated with the spread of HCV (38–40). In recent years, as China’s healthcare system has continuously improved, bloodborne, iatrogenic, and vertical transmission routes have been significantly controlled. However, the number of HCV infections in Xiuyu District remained high, and the epidemic showed clustered characteristics. We speculate that transmission within families from untreated infected individuals and high-risk sexual behaviors may be key factors contributing to this situation.

In our study, we identified one inconsistent sample. Excluding laboratory contamination (the blank control was negative), we considered either a mixed infection with two different genotypes or an intergenotypic recombinant virus. Since the sequencing chromatogram of this sample showed single, uniform peaks, the possibility of mixed infection was considered relatively small (41). This sample exhibited a similar pattern to that of two recombination cases in Heilongjiang Province (42). This observation provides preliminary clues for subsequent analysis of mutation and recombination sites within this strain. In the meantime, this finding suggests that analyzing two HCV genomic fragments can help obtain more accurate genotyping. Additionally, some studies have found that recombinant hepatitis C viruses may exhibit greater drug resistance, and new recombinant strains could pose challenges for HCV treatment (43, 44). HCV genotyping can identify recombinant strains and locate genetic variations, providing a scientific basis for drug development and personalized treatment strategies.

Molecular transmission network analysis can identify potential connections among hepatitis C virus strains and reflect actual transmission relationships between infected individuals (45, 46). It has been commonly used to study transmission characteristics in HIV infections. In recent years, this method has also been applied to investigate HCV transmission dynamics (47). In a transmission network, a greater number of sequences within transmission clusters indicates stronger clustering and a higher risk of transmission (48). In our study, over 60% of the samples were included in the transmission network. This clustering rate exceeded that reported in a similar survey in Guangdong Province (29), indicating close transmission links among HCV-infected individuals in Xiuyu District. The transmission clusters in this study were primarily composed of subtypes 1b and 2a, which formed independent clusters with no observed connections between them. This suggested that the risk of transmission might be higher within the same subtype than between different subtypes (48). Within the transmission clusters, samples with more than 5 edges were predominantly genotype 1b. In the transmission network, samples connected by more edges indicate more complex transmission relationships and a greater likelihood of being high-risk transmitters (49). We recommend focusing monitoring on individuals with a higher number of edges in the subtype 1b cluster to prevent them from becoming super-spreaders.

The largest transmission clusters in both the Core and NS5B fragments consisted of cases infected with subtype 1b, most of whom were older population of Pinghai Town. The network diagram showed that the GT 1b samples had stronger connections than the GT 2a samples, indicating stronger clustering. There will be a potentially higher risk of regionally clustered transmission among the older population infected with subtype 1b in Pinghai Town. The reason for this phenomenon may be that their immune function is relatively weak, making them more susceptible to HCV infection. Moreover, the limited range of daily activities among the older population may facilitate regionally clustered transmission. Additionally, the older population are more likely to seek medical care due to underlying health conditions, which may increase the detection rate of HCV (50, 51). Therefore, the study recommends targeted treatment based on their genotypes. High-risk contacts should be screened, and testing for regular sexual partners should be carried out. At the same time, health education on HCV prevention and control should be provided to the older population, especially promoting condom use to reduce the risk of sexual transmission and lower the incidence of hepatitis C among high-risk groups in the district. Identifying transmission clusters and key populations can help target high-risk groups for screening and intervention, offering a more accurate basis for formulating hepatitis C control strategies.

This study is limited by a small sample size and a lack of behavioral data, which constrains in-depth analysis of disease-related factors. As the sample consists of retrospective cases, findings may not fully reflect current hepatitis C prevalence. Future research should increase sample size and collect detailed epidemiological data, including behavioral patterns, family history, and transmission routes, to better inform hepatitis C prevention and control strategies.

## 5 Conclusion

In conclusion, our study identified locally prevalent HCV strains, mainly genotypes 1b and 2a, in key areas of the southeast coast of China. It suggested that there might be cluster transmission in this district. However, we still need more detailed epidemiological case investigations and experimental data in the future to obtain an accurate estimate of hepatitis C prevalence. Concurrently, we should prioritize providing targeted treatment for all existing HCV carriers in the district. The government and related medical institutions should focus on screening among farmers and increase monitoring of the older population in Pinghai town. These measures will effectively block the chain of viral transmission and create the conditions necessary for the ultimate elimination of hepatitis C.

## Data Availability

The data presented in the study are deposited in the GenBank repository, accession numbers PX917207-PX917304.
