# Supplementary material for: Analysis of genetic evolution and molecular transmission of hepatitis C virus in key areas of the southeast coast of China
Source: Front Public Health. 2026 Mar 4;14:1746631. doi: 10.3389/fpubh.2026.1746631 (PMC12996104; doi:10.3389/fpubh.2026.1746631)
Supplement: Supplementary file 1 [file Data_Sheet_1.zip › Supplementary documents/The Medical Ethics Review Form (Original Version).pdf]

伦理审查意见表（科研项目）

闽疾控伦审（2024）第（001）号

<table>
<tr><td>项目名称</td><td colspan="4">丙型肝炎疾病负担综合评价指标体系构建与实证研究</td></tr>
<tr><td>项目负责人</td><td>连巧龄</td><td>所在单位</td><td colspan="2">福建省疾病预防控制中心</td></tr>
<tr><td>联系电话</td><td>15806039011</td><td>电子邮箱</td><td colspan="2">657422218@qq.com</td></tr>
<tr><td>项目来源</td><td>福建省卫生健康科技计划项目</td><td>项目起止时间</td><td colspan="2">2025.01-2027.12</td></tr>
<tr><td>受理日期</td><td>2024.5.13</td><td>审查日期</td><td>2024.5.14</td><td>2024.5.23</td></tr>
<tr><td>审查方式</td><td colspan="4">☐ 会议审查 ☑简易审查</td></tr>
<tr><td>审查对象</td><td colspan="4">☑ 有关人的涉及生命科学和医学问题的生物样本、信息数据等资料的收集、记录、使用或储存等活动;
☐ 新技术或新产品在人体上进行试验的研究活动;
☐ 对人的生理、心理行为、病理现象、疾病病因和发病机制，以及疾病预防、诊断、治疗或康复等进行的研究活动;
☐ 对人的生殖、生长、发育、衰老等进行的研究活动。</td></tr>
<tr><td>审查意见</td><td colspan="4">☐ 批准 ☑ 做必要修改后批准 ☐ 修改后再审
☐ 不批准 ☐ 继续研究 ☐ 暂停或终止研究</td></tr>
<tr><td>跟踪审查频次</td><td colspan="2">☐ 定期: ____年/月</td><td colspan="2">☑ 不定期</td></tr>
<tr><td colspan="5">审查单位（盖章）：福建省疾病预防控制中心医学伦理委员会
主任委员或副主任委员（签字）：[illegible]
日期：2024.5.24
（印章：福建省疾病预防控制中心 医学伦理委员会）</td></tr>
</table>

夸克扫描王
极速扫描，就是高效
