# Supplementary material for: Analysis of genetic evolution and molecular transmission of hepatitis C virus in key areas of the southeast coast of China
Source: Front Public Health. 2026 Mar 4;14:1746631. doi: 10.3389/fpubh.2026.1746631 (PMC12996104; doi:10.3389/fpubh.2026.1746631)
Supplement: Supplementary file 1 [file Data_Sheet_1.zip › Supplementary documents/The Medical Ethics Review Form (Translated Version).docx]

Document number: AF/SC-04/01.0

Ethical Review Form (Research Project)

Ethical Review of the Fujian Provincial Center for Disease Control and Prevention (2024) No. (001)

| Project name | Construction and Empirical Study of Comprehensive Evaluation Index System of Disease Burden of Hepatitis C | | | |
| --- | --- | --- | --- | --- |
| Project leader | Lian Qiaoling | Organization | Fujian Provincial Center for Disease Control and Prevention | |
| Contact number | 15806039011 | E-mail | 657422218@qq.com | |
| Project source | Fujian Province Health Science and Technology Planning Project | Project start and end dates | 2025.01-2027.12 | |
| Date of acceptance | 2024.5.13 | Review date | 2024.5.14 | 2024.5.23 |
| Review method | □ Meeting review ☑ Simplified review | | | |
| Review subject | ☑ Activities involving the collection, recording, use or storage of biological samples, information data and other materials related to human beings in the fields of life sciences and medicine;  □Research activities involving the testing of new technologies or products on humans;  □Research activities on human physiological and psychological behaviors, pathological phenomena, causes and mechanisms of diseases, as well as disease prevention, diagnosis, treatment or rehabilitation;  □Research activities on human reproduction, growth, development and aging. | | | |
| Review comments | □Approval ☑ Approve after making necessary modifications □ Re-examine after modifications  □No approval □ Continue the study □ Suspend or terminate the study | | | |
| Frequency of tracking reviews | □Regular: Year/Month | | ☑ Irregularly scheduled | |
| Review Unit (Seal): Medical Ethics Committee of Fujian Provincial Center for Disease Control and Prevention  The Chairperson or Vice-Chairperson(Signature):  Date : | | | | |
